# TBK1 Mutations in Amyotrophic Lateral Sclerosis and Frontotemporal Dementia: Mechanistic Insights into Impaired Autophagy and Proteostatic Failure

**DOI:** 10.3390/cells15050477

**Published:** 2026-03-06

**Authors:** Francesca Manganelli, Camilla Perfetto, Olga Carletta, Valeria Gerbino

**Affiliations:** 1 IRCSS Fondazione Santa Lucia, European Center for Brain Research (CERC), Via del Fosso di Fiorano 64, 00143 Rome, Italy; 2 Graduate Program in Cell and Molecular Biology, Department of Biology, University of Rome Tor Vergata, 00133 Rome, Italy; 3 Graduate Program in Science and Biomedical Technologies, Department of Science, University “Roma Tre”, 00146 Rome, Italy; 4 Institute of Translational Pharmacology, National Research Council (CNR), 00133 Rome, Italy; 5 Zuckerman Institute of Mind Brain and Behavior, Columbia University, New York, NY 10032, USA

**Keywords:** Amyotrophic Lateral Sclerosis (ALS), frontotemporal dementia (FTD), autophagy, TANK-binding kinase 1 (TBK1)

## Abstract

Mutations in the TANK-binding kinase 1 (TBK1) gene represent a significant genetic link across the Amyotrophic Lateral Sclerosis (ALS) and Frontotemporal Dementia (FTD) spectrum. As a multifunctional serine/threonine kinase, TBK1 serves as a central orchestrator of the autophagy–lysosome pathway, regulating critical stages from initial cargo recognition and autophagosome biogenesis to vesicle maturation and lysosomal fusion. This review examines the mechanisms by which TBK1 coordinates these diverse autophagic functions. We then focus on how ALS/FTD-associated mutations—ranging from truncating variants causing haploinsufficiency to domain-specific missense mutations—disrupt these essential processes.

## 1. Amyotrophic Lateral Sclerosis and Frontotemporal Dementia

Amyotrophic lateral sclerosis (ALS) and frontotemporal dementia (FTD) represent two ends of a shared neurodegenerative disease spectrum.

ALS is characterized by motor neuron loss, neuromuscular denervation, and progressive paralysis, including paralysis of the respiratory muscles, which results in death from respiratory failure. Approximately 10% of ALS cases are familial (fALS), while the remaining 90% occur sporadically (sALS). Median survival is 2 to 4 years from onset, with only 5–10% of patients surviving beyond 10 years [1,2]. FTD is the second most common early-onset dementia. FTD involves focal atrophy of the frontal and anterior temporal lobes, manifesting as profound changes in personality, social conduct, and language [3,4,5].

The genetic architecture of the ALS/FTD spectrum is characterized by a high degree of phenotypic pleiotropy, where mutations in a single gene can result in clinical presentations ranging from pure motor neuron disease to dementia. The most significant link in this spectrum is the hexanucleotide repeat expansion in *C9orf72,* which stands as the leading genetic cause for both conditions, identified in approximately 30–40% of familial ALS and 25% of familial FTD cases [6]. Beyond *C9orf72*, several other genes reinforce this overlap, with *TARDBP*, *SQSTM1*, *VCP*, *FUS*, *TBK1*, and *CHCHD10* being critical genetic players in these neurological disorders [6].

ALS and FTD also remarkably share molecular and histopathological features. The most significant pathological overlap between ALS and FTD involves aggregation of TAR DNA-binding protein 43 (TDP-43) [7]. In fact, ubiquitinated and phosphorylated neuronal and glial cytoplasmic aggregates of the TDP-43 protein in the motor cortex and in the spinal cord are the histopathological hallmark in 97% of ALS cases. TDP-43 pathology is also found in 50% of FTD, most commonly associated with the clinical syndrome of behavioral variant FTD (bvFTD), which has clinical overlap with ALS [8].

These TDP43-positive inclusions can be substrates for autophagy [9,10], suggesting that accumulation of TDP-43 protein aggregates in ALS/FTD patients could be a result of impaired autophagy. In further support of this hypothesis, genetic evidence has also connected autophagy to ALS and FTD, as several genes implicated in autophagy are found to be mutated in ALS/FTD patients [11]. Among them, *TANK-Binding Kinase 1* (*TBK1*) is the primary gene of interest in this review. *TBK1* is a major genetic contributor to both ALS and FTD [12,13,14,15,16,17,18,19], and a key player in the autophagy pathway.

We will examine the role of TBK1 in autophagy, with a particular focus on its involvement in the pathogenesis of ALS and FTD.

## 2. Autophagy

Autophagy is an evolutionarily conserved degradative process essential for maintaining cellular proteostasis by recycling damaged organelles and misfolded proteins. Macroautophagy (hereafter referred to as autophagy) involves the de novo synthesis of double-membrane vesicles, termed autophagosomes, which sequester cytoplasmic cargo for lysosomal degradation. Selective autophagy is a specific form of autophagy that uses specialized receptor proteins to target specific cargo for degradation.

Targets of selective autophagy include protein aggregates (aggrephagy), mitochondria (mitophagy), peroxisomes (pexophagy), endoplasmic reticulum (ER-phagy), lipid droplets (lipophagy), and pathogens (xenophagy). Neurons rely heavily on selective autophagy to clear specific toxic substrates.

**Figure 1:**
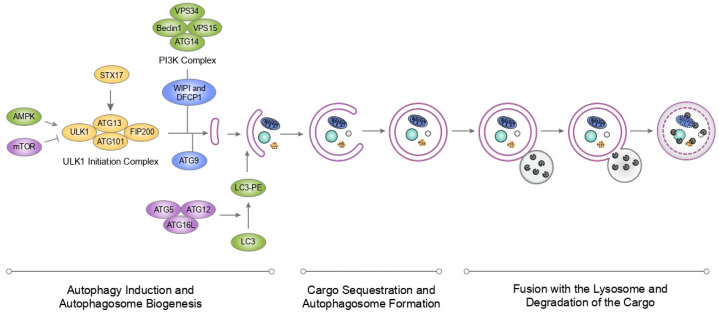
The Core Molecular Machinery of Macroautophagy. Autophagy initiates via a nutrient-sensing rheostat where AMPK activates and mTORC1 inhibits the ULK1 complex (ULK1-ATG13-FIP200-ATG101). Activated ULK1 recruits ATG9A-positive vesicles and phosphorylates the Class III PI3K complex (BECN1-ATG14-VPS15-VPS34) to generate PI3P at the isolation membrane. This PI3P signal recruits WIPI2 and the ATG12-ATG5-ATG16L1 complex, which functions as an E3 ligase to catalyze the conjugation of LC3-I to phosphatidylethanolamine (PE). The resulting lipidated LC3-II facilitates phagophore expansion and cargo sequestration. Following vesicle closure, the autophagosome fuses with the lysosome. Finally, lysosomal hydrolases degrade the internal cargo.

From a mechanistic point of view, autophagy can be delineated into three principal stages: (1) induction and autophagosome biogenesis, (2) selective cargo sequestration and autophagosome maturation, and (3) fusion with the lysosome and degradation of the cargo (Figure 1). Each stage is tightly regulated in a sequential manner by the core autophagic machinery, comprising Autophagy-related (Atg) proteins, which were initially identified in yeast [20] and are highly conserved across eukaryotic evolution.

The initiation of autophagy is governed by mTORC1 and AMPK. Under nutrient-depletion conditions, mTORC1 maintains cellular growth by phosphorylating and inhibiting the Unc-51-like kinase 1 (ULK1) complex (comprising ULK1, ATG13, FIP200, and ATG101). Conversely, during metabolic stress or starvation, the activation of AMPK directly inhibits mTORC1 and simultaneously activates ULK1 via phosphorylation at specific residues (e.g., Ser317 and Ser777) [21,22]. This activation triggers the recruitment of ATG9A-containing vesicles, which act as the initial lipid templates for the developing phagophore [23,24,25] (Figure 1).

Once autophagy is initiated, the ULK1 complex phosphorylates the Class III PI3K complex I (PI3KC3-C1), which includes Beclin1, ATG14, VPS15 and VPS34. Activation of the PI3KC3-C1 generates phosphatidylinositol 3-phosphate (PI3P), which recruits the PI3P-binding proteins double FYVE-containing protein 1 (DFCP1) and WD-repeat protein interacting with phosphoinositides 1/2 (WIPI1/2) to the growing phagophore (Figure 1).

WIPI2 then recruits the ATG12-ATG5-ATG16L complex to the growing phagophore [26]. The ATG12-ATG5-ATG16L complex serves as the E3 ligase necessary for the conjugation of LC3 to phosphatidylethanolamine (PE) on the developing autophagosomal membrane (Figure 1). LC3 governs vesicle elongation, maturation, and the eventual fusion of the autophagosome with the lysosome. Under basal conditions, LC3 exists in a diffuse state distributed between the cytoplasm and the nucleus, referred to as LC3-I. Upon the triggering of autophagy, LC3 undergoes cytoplasmic cleavage by ATG4B [27] and subsequent conjugation to PE, allowing membrane expansion and autophagosome maturation [28,29]. The resulting lipidated form, LC3-II, populates both the internal and external surfaces of the isolation membrane and the mature autophagosome.

Upon closure, the double-membrane autophagosome encapsulates the cargo. The outer membrane subsequently fuses with lysosomes, forming an autolysosome [22]. The trafficking of the autophagosome to the lysosome is a fusion-dependent mechanism that involves regulation by STX17 [30,31], and RAB7 [32]. Because LC3-II is present on both membrane leaflets, the pool exposed to the lysosomal lumen is degraded alongside the sequestered cargo by lysosomal hydrolases. Conversely, the LC3-II localized on the external membrane is cleaved from its PE anchor by ATG4B and recycled for further use [27]. Within the autolysosome, hydrolases degrade the sequestered material into basic metabolites such as amino acids, lipids, and nucleotides, which are recycled back to the cytoplasm [22,33] (Figure 1).

The precision of the selective autophagy process is mediated by autophagy receptors. These receptors function as molecular bridges, utilizing a ubiquitin-binding domain (UBD) to recognize ubiquitinated cargo and an LC3-interacting region (LIR) motif to tether that cargo to the expanding autophagosome membrane [34,35]. The interaction between autophagy receptors and the protein FIP200 has been shown to promote autophagosome biogenesis directly at the sites of cargo recruitment [36,37,38]. Autophagy receptors are regulated at multiple levels to ensure proper timing and specificity of substrate degradation. Phosphorylation is one of the most prominent regulatory mechanisms [39].

## 3. The Pleiotropic Functions of TBK1 in Autophagy: One Kinase, Many Roles

TBK1, also called NF-κB-activating kinase (NAK) or tumor necrosis factor (TNF) receptor-associated factor 2 (TRAF2)-associated kinase (T2K), is a serine/threonine kinase that plays a crucial role in various cellular processes, including the innate immune response, autophagy, and cell proliferation. It was first identified by David Baltimore in the context of its interaction with the adaptor protein TRAF family member-associated NF-κB activator (TANK) [40] and has since been extensively studied for its involvement in signaling pathways related to immune defense, autophagy and disease.

TBK1 is a member of the IκB kinase (IKK) family, sharing structural similarities with IKKε [41,42,43]. It consists of several distinct domains, each contributing to its functional capabilities. The N-terminal Kinase Domain (KD) is responsible for the enzyme’s catalytic activity, facilitating the transfer of phosphate groups to substrate proteins. Moreover, the KD plays an essential role in substrate recognition [43]. The Ubiquitin-like Domain (ULD), positioned adjacent to the kinase domain, is involved in protein–protein interactions essential for TBK1’s function in signaling complexes. The integrity of the ULD is not only crucial for kinase activity but also contributes to substrate specificity [44]. The Scaffold Dimerization Domain (SDD) facilitates oligomerization and interactions with adaptor proteins. The C-terminal Domain (CTD) mediates the recruitment of TBK1 to various signaling complexes, positioning it to phosphorylate downstream effectors [41,42,45].

Upon recruitment to the signaling complex, TBK1 undergoes autophosphorylation at serine 172 (S172) within its kinase domain, a modification essential for its catalytic activation [43,46]. It has also been suggested that upstream kinase(s) can be responsible for the initial phosphorylation of TBK1 at S172 [47,48]. Once active, TBK1 is able to phosphorylate downstream targets.

### 3.1. Phosphorylation of Autophagy Receptors

The first role of TBK1 in autophagy to be discovered was the phosphorylation of autophagy receptors. TBK1 phosphorylates autophagy receptors such as Optineurin, p62/SQSTM1, and NDP52, enhancing their ability to bind ubiquitinated cargo and recruit autophagic machinery [49,50,51,52]. It has been shown that TBK1 also phosphorylates LC3/GABARAP proteins, increasing their stabilization [53].

#### 3.1.1. Optineurin (OPTN)

OPTN acts as an autophagy receptor that selectively targets ubiquitinated cargo (damaged mitochondria, invading pathogens, protein aggregates) for autophagic degradation. It contains a UBAN domain and a zinc-finger domain that bind K63-linked polyubiquitin chains, enabling cargo recognition and bridging to autophagosomal membranes via its LC3-interacting region (LIR). OPTN was first identified as an autophagy receptor in 2011 by Ivan Dikic’s group [49]. In that study, the authors demonstrated for the first time that TBK1 phosphorylated OPTN on S177, enhancing LC3 binding affinity and autophagic clearance of invading bacteria (xenophagy). Subsequently, several additional TBK1-phosphorylation sites on OPTN were identified by the same group [52], including S473 and S513. In addition to promoting LC3 affinity, TBK1-mediated phosphorylation of OPTN also promotes mitochondrial recruitment of the autophagic cargo by enhancing the binding of OPTN to Ubiquitin chains [52]. Additional studies by Lazarou and colleagues [54] and by Heo and colleagues [51] demonstrated that the TBK1-mediated phosphorylation of OPTN also increases its binding to ubiquitin chains decorating the autophagic cargo, thus facilitating cargo clearance.

In 2014, OPTN was identified as a mitophagy receptor by the Holzbaur group [55]. Furthermore, TBK1 was shown to be recruited to damaged mitochondria in a OPTN’s dependent manner [50], thus forming a positive feedback loop: OPTN clusters TBK1, TBK1 phosphorylates OPTN, and phosphorylated OPTN binds LC3 more tightly and stabilizes the mitophagy machinery [56,57]. More recently, TBK1-mediated phosphorylation of OPTN has also been linked to the selective degradation of damaged lysosomes [58]

#### 3.1.2. p62

p62 was the first discovered selective autophagy receptor [59,60] and represents a central focus in ALS and FTD research, due to its presence in neuronal protein aggregates discovered in post mortem tissues from patients [61].

To facilitate selective autophagy, p62 relies on two critical motifs: the UBA (Ubiquitin-Associated) Domain, located at the C-terminus, and the LIR (LC3-Interacting Region) Motif. While the first binds to K63- and K48-linked polyubiquitinated chains on cargo [62], the latter facilitates the docking of the p62-cargo complex onto the LC3-II proteins anchored in the growing phagophore [60,63]. Under basal conditions, p62 exists in a dimerized, relatively inactive state. TBK1 acts as a critical switch for p62 activity. It has in fact been shown that by phosphorylating p62 at Ser403 (within the UBA domain), TBK1 increases p62’s affinity for polyubiquitinated chains [64], thus enhancing the clearance of the autophagic cargo.

#### 3.1.3. NDP52

NDP52 is both an autophagy receptor and a TBK1 adaptor. It binds ubiquitinated cargo and recruits TBK1 through the adaptors NAP1 and SINTBAD, promoting mitophagy and xenophagy.

The role of NDP52 in selective autophagy was first pioneered in the context of xenophagy, where it was shown to restrict bacterial growth by targeting ubiquitin-coated pathogens to the autophagosome [65]. This early work identified the essential recruitment of TBK1 to NDP52-positive sites, a discovery that laid the groundwork for our current understanding of how the NDP52-TBK1 axis orchestrates the clearance of both pathogens and neurotoxic aggregates. More recently, NDP52 was also described as a primary orchestrator of autophagosome biogenesis. NDP52 facilitates the direct delivery of the ULK1 complex to ubiquitinated cargo by interacting with FIP200, thereby initiating in situ autophagosome formation. TBK1 is indispensable to this process: it phosphorylates NDP52 to enhance its ubiquitin-binding affinity and strengthens the structural association between NDP52 and FIP200. This TBK1-dependent recruitment creates a high-efficiency ‘trigger’ for selective autophagy, ensuring that neurotoxic substrates are rapidly sequestered by the autophagic machinery [37].

#### 3.1.4. TAX1BP1

TAX1BP1 is an autophagy receptor with known roles in xenophagy [66], and aggrephagy [67]. More recently, the functional interaction between TBK1 and TAX1BP has also been highlighted in lysophagy. During lysophagy, TBK1 interacts with TAX1BP1. This complex is recruited to ubiquitinated lysosomal membranes. TBK1 then recruits FIP200, a member of the ULK1 initiation complex, directly to the damaged lysosome to begin de novo autophagosome formation around the organelle [68]. Another study has also identified a TBK1-FBXO3-TMEM192 axis in which TBK1 phosphorylates the E3 ligase component FBXO3, which then ubiquitinates the lysosomal membrane protein TMEM192, creating the “eat me” signal required for TAX1BP1 recruitment on damaged lysosomes [69].

### 3.2. Role of TBK1 in Autophagy Initiation and Autophagosome Formation

Because most of the early work on TBK1 focused on its role in selective autophagy (e.g., phosphorylation of cargo receptors like OPTN, p62, NDP52), it was long thought to act exclusively at the cargo-recognition stage. Over the last decade, multiple studies have provided direct evidence that—beyond cargo sequestration—TBK1 serves as a critical coordinator of autophagosome maturation, ensuring the efficient transition from a nascent double-membrane vesicle to a degradative autolysosome.

The role of TBK1 in autophagy initiation was first highlighted in the context of xenophagy, where it facilitates the recruitment of WIPI2 to invading bacteria [70]. This initiation capacity extends to the phosphorylation of Syntaxin 17 (Stx17) at Serine 202. In fact, this specific modification triggers the translocation of Stx17 from the Golgi apparatus to the mammalian pre-autophagosomal structure (mPAS). Once localized, phospho-Stx17 interacts with ATG13 and FIP200, thereby scaffolding the mPAS assembly during nutrient deprivation [71].

TBK1 also governs the supply of membranes required for vesicle elongation. By phosphorylating RAB7A at S72, TBK1 promotes the recruitment of ATG9A-positive vesicles—derived from the Golgi—to damaged mitochondria [72]. These ATG9A vesicles are essential membrane sources that support phagophore nucleation alongside the PI3K complex and WIPI proteins.

**Figure 2:**
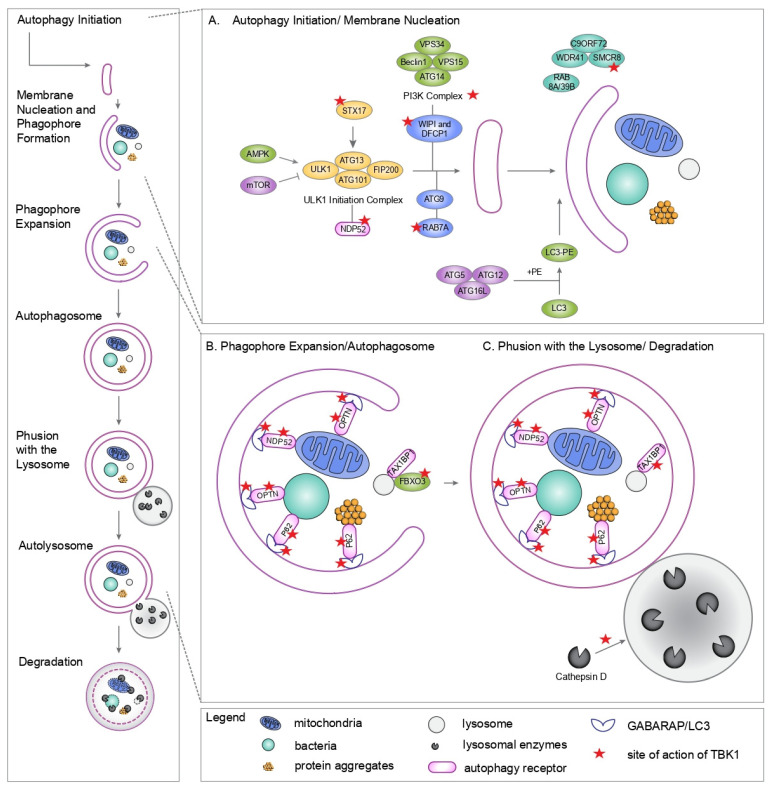
Distinct TBK1 roles at different steps in the autophagy pathway. (**A**) Autophagy Induction and Autophagosome Biogenesis: (1) TBK1 phosphorylates STX17, enhancing the initial steps of membrane nucleation and phagophore formation; (2) TBK1 stabilizes the recruitment of WIPI2 to nascent autophagosomes; (3) TBK1 facilitates the indirect recruitment of ULK1 to damaged cargo via binding to the PI3K Complex; (4) TBK1 promotes the association of NDP52 with the ULK1 complex, facilitating the activation of ULK1 directly on the cargo; (5) TBK1 phosphorylates Rab7a, promoting recruitment of ATG9 vesicles to cargos; (6) TBK1 phosphorylates SMCR8 in the C9ORF72/WDR41/SMCR8 complex, which functions as a GDP/GTP exchange factor for Rab8a and Rab39, thereby controlling autophagic flux. (**B**) Phagophore Expansion and Autophagosome: (1) TBK1 phosphorylates autophagy receptors (OPTN, P62, NDP52) enhancing their binding to the cargo and to LC3 proteins during mitophagy, aggrephagy, xenophagy and lysophagy; (2) TBK1 phosphorylates LC3 and GABARAPL2, at S93/96 and S87/88 respectively, to impede premature cleavage of LC3s from nascent autophagosomes by ATG4; (3) TBK1 phosphorylates FBXO3, which binds to the lysosomal membrane protein TMEM192 and to the autophagy adaptor TAX1BP1, enhancing lysophagy. (**C**) Lysosome and Degradation: TBK1 delivers Cathepsin D to the autophagolysosomal compartment.

Beyond membrane trafficking, TBK1 may regulate the ULK1 complex indirectly through the phosphorylation of SMCR8. SMCR8 forms a heterotrimeric complex with WDR41 and C9ORF72, which functions as a ULK1 interactor and a Guanine Nucleotide Exchange Factor for RAB8a and RAB39b [73]. While the precise regulatory impact of TBK1-mediated SMCR8 phosphorylation on ULK1 activity remains a subject of ongoing investigation, it suggests a sophisticated layer of feedback between TBK1 and the core autophagy machinery (Figure 2).

In specialized pathways like OPTN-mediated mitophagy, TBK1 displays functional redundancy with the ULK1/2 kinases. Here, TBK1 directly tethers the PI3K complex to the site of organelle damage, ensuring that autophagosome construction is physically coupled to the cargo rather than occurring stochastically within the cytoplasm [74]. This localized initiation is further supported by the interaction between TBK1 and the receptor NDP52, which recruits the ULK1 complex to ubiquitinated substrates [38]. Notably, this NDP52-TBK1-ULK1 axis operates independently of traditional nutrient sensors like AMPK or mTOR, representing a specialized, cargo-driven bypass of the standard initiation hierarchy [37].

### 3.3. Role of TBK1 in Autophagosome Maturation and Fusion with the Lysosome

The influence of TBK1 extends into the terminal stages of the pathway. TBK1 interacts with Rab8b to facilitate the transport and fusion of autophagosomes with lysosomes. While TBK1 deficiency may not completely halt initial vesicle formation, it results in the accumulation of stalled, incomplete autophagic structures, indicating a failure in maturation [75]. This maturation is supported by several biochemical mechanisms. First, TBK1 phosphorylates surface-exposed serine residues on LC3C (S93/S96) and GABARAP-L2 (S87/S88). This stabilizes the lipidated coat of these modifiers, ensuring the structural integrity of the growing vesicle and maintaining unidirectional flow toward the lysosome [53]. Second, TBK1 aids in the delivery of the lysosomal hydrolase cathepsin D, which is vital for the degradative capacity of the autolysosome [75] (Figure 2).

## 4. The Impact of ALS/FTD-Associated TBK1 Mutations on Autophagy—Lessons from In Vitro and In Vivo Models

The highly conserved autophagy pathway is essential for maintaining protein homeostasis and mitigating the neurodegenerative insults that drive ALS and FTD. By facilitating the clearance of misfolded and toxic proteins, TBK1 plays a pivotal role in preserving neuronal health throughout an organism’s lifespan. Consequently, mutations in the *TBK1* gene disrupt these regulatory mechanisms, leading to the accumulation of protein aggregates that characterize the neurodegenerative process.

The frequency of *TBK1* mutations varies depending on the clinical presentation (ALS, FTD, or both) and the geographic population studied. Generally, it is considered a moderate-frequency gene [6]. While percentages fluctuate between cohorts, the consensus indicates an estimated frequency of 1–4% in fALS and less than 1% in sALS [13,16]. In familial FTD, *TBK1* mutations have a 1–2% frequency, with the highest frequency (3–4%) in families where both FTD and ALS are present, further highlighting its role as a key gene at the crossroads of the two diseases [13,14,15,76].

A common pathological hallmark of ALS and FTD patients bearing *TBK1* mutations is the presence of neuronal and glial TDP43 and/or p62 positive aggregates in brain and spinal cord tissues [15,77,78]. This observation warrants further investigation into the roles of TBK1 in the aggregate-clearing pathways: autophagy and the ubiquitin-proteasome system.

*TBK1* mutations linked to FTD and ALS are mostly heterozygous loss-of-function variants, including nonsense, frameshift, and splice-site mutations, which result in haploinsufficiency and reduced kinase activity [13,15,79,80]. Truncating mutations often lead to nonsense-mediated decay of TBK1 mRNA or unstable protein products, effectively diminishing TBK1 function [13]. Beyond simple loss of protein levels, later biochemical work demonstrated that many missense mutations diminish TBK1’s ability to autophosphorylate at Ser172 and modify target proteins, even when total TBK1 levels remain normal [79,80]. The impact of generic TBK1 loss of function on autophagy in the context of ALS has been investigated initially through genetic knock-down or knock-out of the *TBK1* gene [81,82]. With the aim of highlighting the potential contribution of specific TBK1-ALS mutations to disrupted proteostasis during the neurodegenerative disease process, this review will focus specifically on studies describing the effects of *TBK1* mutations known to be associated with ALS/FTD.

The functional impact of these mutations has been validated across various cellular models. De Majo et al. found that the variant G217R in the KD and the truncation R357X in the ULD both abolish OPTN phosphorylation, effectively mimicking a kinase-dead state [80]. Similarly, the KD mutant G175S was found to impair p62 phosphorylation and the degradation of TDP-43 [83]. Ye et al. systematically tested 25 missense variants in TBK1-knockout cells, finding that mutations in the KD (such as R25H, R47H, N129D, V132E, G217R, R228H) showed abolished or markedly reduced OPTN Ser177 and p62 S403 phosphorylation [79]. Some mutations in the ULD and SDDs (such as I450K and M559R) also showed markedly reduced activity, while a subset of mutants exhibited substrate-specific defects—retaining their ability to phosphorylate autophagy receptors despite losing their activity toward other substrates like IRF3 [79].

These biochemical deficits translate directly to cellular pathology. For example, iPSC-derived motor neurons from patients carrying the TBK1 T77WfsX4, E643del or the Y185X mutations display an accumulation of p62 aggregates and disrupted autophagosome elongation [84]. Furthermore, in 2021, the Holzbaur group examined a panel of ALS/FTD-associated missense mutants in primary hippocampal neurons. They found that TBK1 mutations, which strongly reduce kinase activity or dimerization, lead to impaired OPTN Ser177 phosphorylation on damaged mitochondria and slower mitophagic flux [85].

Moving beyond in vitro assays, in 2020, the first mouse models bearing TBK1 mutations associated with ALS were generated [82]. Specifically, CRISPR-Cas9 was used to insert either the TBK1 R228H mutation or the TBK1 G217R mutation in mice [82]. In spinal cord in vivo, TBK1 was found to be phosphorylated (and therefore active) in ubiquitinated protein aggregates that were targeted to autophagy in motor neurons of mice bearing the well-studied SOD1 G93A mutation [82]. Mice bearing either the TBK1 R228H or the TBK1 G217R mutation, in combination with the SOD1 G93A mutation, showed impaired phosphorylation of p62 and OPTN and increased accumulation of SOD1 protein aggregates in motor neurons. This autophagy defect correlated with cell stress, disruption of the Golgi apparatus, and premature motor neuron death in TBK1 R228H; SOD1 G93A mice compared to TBK1 WT; SOD1 G93A mice [82]. Interestingly, a couple of years later, another group showed that TBK1 was similarly activated in DPR aggregates in an (AAV)-based C9orf72 mouse model of C9ORF72 FTD/ALS [86]. The authors found that combining the TBK1 R228H mutation with the C9orf72 repeat expansion increases the accumulation of poly(GA) inclusions and exacerbates endosomal defects in C9orf72 mice. This resulted in accelerated neurodegeneration and reduced motor performance of poly(GA) in mice [86]. These two independent studies demonstrated a key role for TBK1 kinase activity in mediating proteostasis and maintaining neuronal health in vivo.

Finally, the TBK1 E696K mutation (in the CTD) highlights the importance of TBK1 protein–protein interactions. The E696K mutation does not abolish catalytic activity but selectively disrupts the interaction between TBK1 and OPTN [13]. In cellular mitophagy assays, E696K TBK1 is poorly recruited to damaged mitochondria, shows reduced OPTN Ser177 phosphorylation in situ, and fails to support efficient mitophagic ring formation [50,85]. A 2024 knock-in mouse study confirmed that E696K causes selective loss of OPTN binding, but did not lead to diminished OPTN phosphorylation in vivo [87]. Nevertheless, neurons from aging (19-month-old) TBK1 E696K mice showed increased accumulation of p62 and GABARAPL1-positive inclusions. In the spinal motor neurons of mutant mice, the authors found a significant build-up of enlarged LAMP1-positive lysosomes, suggesting dysfunctional or incomplete lysosomal degradation [87]. A recent phosphoproteomic study of human iPSC-derived neurons confirmed that the TBK1 E696K mutation reduces phosphorylation of known TBK1 targets in the autophagy pathway (including OPTN, p62, GABARAPL2 and RAB7A) [88]. These studies, together, highlight the importance of TBK1’s CTD and the relevance of TBK1 protein–protein interactions—beyond kinase activity-on healthy neuronal autophagy in both mouse models and human neurons.

Ultimately, these multi-model findings establish that whether through haploinsufficiency, diminished kinase activity, or disrupted protein–protein interactions, *TBK1* mutations converge on a common pathogenic mechanism: the failure of autophagic clearance that precipitates proteostatic collapse and accelerated neurodegeneration in ALS and FTD.

## 5. Discussion and Conclusions

**Figure 3:**
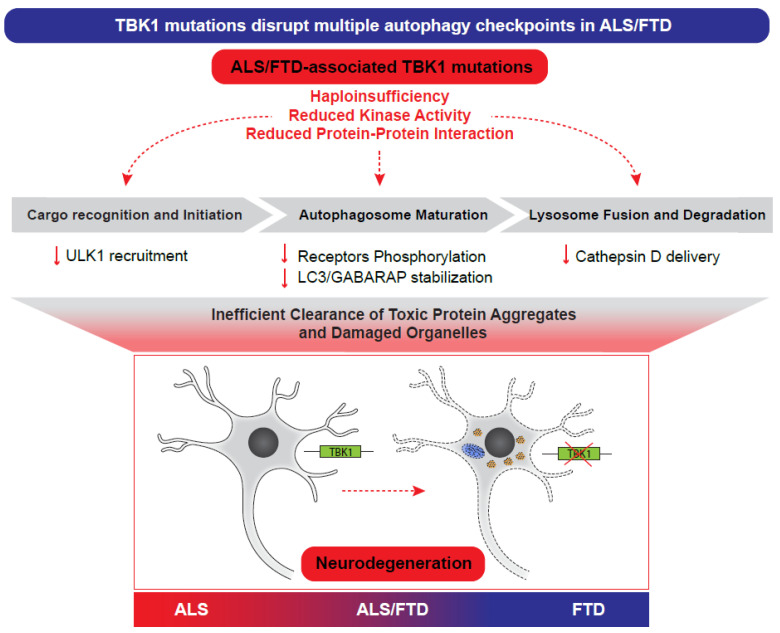
*TBK1* mutations disrupt multiple autophagy checkpoints in ALS/FTD. Graphical summary of the effects of *TBK1* ALS/FTD mutations on the autophagy pathway, and the functional consequences in neurons (accumulation of damaged mitochondria and toxic protein aggregates).

Increasing genetic and mechanistic studies position TBK1 as a pivotal regulator of selective autophagy whose dysfunction contributes directly to ALS/FTD pathogenesis. Rather than acting at a single checkpoint, TBK1 coordinates multiple stages of autophagy—cargo recognition, autophagosome biogenesis, and maturation—thereby linking ubiquitin-tagged substrates to their efficient lysosomal clearance. This multifunctional role provides a unifying framework for understanding why TBK1 loss of function predisposes neurons to degeneration (Figure 3).

The exceptional vulnerability of neurons to TBK1 dysfunction reflects their unique reliance on selective autophagy for lifelong maintenance of protein and organelle quality [89]. TBK1 mutations disrupt phosphorylation of OPTN, p62, NDP52, and LC3/GABARAP proteins, impairing mitophagy, aggregate clearance, and autophagosome maturation. In vivo, these defects manifest as p62-positive inclusions, enlarged lysosomes, and accumulation of disease-relevant substrates such as TDP-43, SOD1 and C9orf72-derived dipeptide repeats. Beyond these well-established roles in phosphorylating selective autophagy receptors, a key conceptual advance of recent years is the recognition that TBK1 also operates upstream of autophagy initiation. By stabilizing interactions between receptors such as NDP52 and the ULK1–FIP200 complex, TBK1 enables a cargo-driven mode of autophagy initiation that is spatially restricted and independent of canonical nutrient-sensing pathways. This mechanism is likely critical in neurons, where long axonal processes and localized damage necessitate precise, on-site autophagosome formation. Consequently, ALS/FTD-associated *TBK1* mutations impair not only receptor activation but also the earliest steps of selective autophagy, leading to cumulative proteostatic stress over time (Figure 3).

Accumulating evidence indicates that the same *TBK1* mutation can give rise to ALS, FTD, or a mixed phenotype in different individuals, including within the same family [14,90], arguing strongly against a mutation-specific, deterministic clinical outcome. Instead, a likely model is that TBK1 loss establishes a common upstream vulnerability state, while patient-specific genetic background and additional biological risk factors act as modifiers that bias downstream network failure toward motor or frontotemporal systems. In this context, variation in genes and pathways involved in proteostasis, mitochondrial homeostasis, vesicle trafficking, and neuroinflammatory regulation is likely to shape how strongly TBK1-dependent defects are expressed in distinct neuronal populations. Although individual mutations may differentially affect TBK1 stability or its interactions with autophagy adaptor proteins and thereby modulate the degree of pathway impairment, these differences largely scale a common biological deficit rather than redirect it toward disease-specific mechanisms. Consequently, ALS, FTD, and ALS/FTD linked to TBK1 are best viewed as a phenotypic spectrum emerging from shared upstream dysfunction, with the dominant clinical presentation determined by the interaction between this core molecular defect and patient-specific genetic and cellular context, and by which vulnerable neural systems first cross a threshold of failure.

The complexity of TBK1 biology extends beyond autophagy. Although this review has focused on autophagy, TBK1’s established role in innate immune signaling raises the possibility that autophagic and inflammatory dysfunction synergize to drive neurodegeneration. From a therapeutic perspective, TBK1-associated ALS/FTD highlights both opportunity and complexity. Direct kinase activation may restore selective autophagy in haplo-insufficient states but risks exacerbating inflammatory signaling. Alternatively, targeting downstream autophagy nodes, enhancing compensatory kinases such as ULK1, may provide safer strategies. Ultimately, mutation-specific and/or cell-specific interventions will likely be required.

In conclusion, TBK1 emerges as a central organizer of selective autophagy whose disruption compromises neuronal proteostasis at multiple levels. Defining how distinct *TBK1* mutations perturb autophagy in space and time is critical for understanding ALS/FTD pathogenesis and for designing rational, mechanism-based therapies.

## Data Availability

No new data were created or analyzed in this study.

## Abbreviations

ALS	Amyotrophic Lateral Sclerosis
AMPK	AMP-Activated Protein Kinase
ATG	Autophagy-related
bvFTD	Behavioral variant FTD
*C9Orf72*	*Chromosome 9 Open Reading Frame 72*
*CHCHD10*	*Coiled-coil-helix-coiled-coil helix domain10*
CTD	C-Terminal Domain
DFCP1	Double FYVE-Containing Protein 1
DPR	Dipeptide Repeat
fALS	Familial ALS
*FBXO3*	F-box only protein 3
FIP200	FAK family-Interacting Protein of 200 kDa
FTD	Frontotemporal Dementia
GABARAP	γ-Aminobutyric Acid Receptor-Associated Protein
IKKε	Inhibitor of nuclear factor kappa-B kinase subunit epsilon
KD	Kinase Domain
LAMP1	Lysosome-Associated Membrane Protein 1
LC3	Microtubule-associated protein 1 light chain 3
LIR	LC3-Interacting Region
mPAS	Mammalian pre-autophagosomal structure
mTOR	Mammalian Target of Rapamycin
mTORC1	Mechanistic Target of Rapamycin Complex 1
NAK	NF-κB-Activating Kinase
NAP1	Nucleosome Assembly Protein 1
OPTN	Optineurin
PE	Phosphatidylethanolamine
PI3KC3-C1	Class III PI3K Complex I
PI3P	Phosphatidylinositol-3-Phosphate
PINK1	PTEN-Induced Kinase 1
RAB	Ras-related protein
sALS	Sporadic ALS
SDD	Scaffold Dimerization Domain
SINTBAD	Similar to NAP1 TBK1 Adaptor Domain
SMCR8	Smith-Magenis Syndrome Chromosomal Region Candidate Gene 8
SQSTM1/p62	Sequestosome 1
STX17	Syntaxin 17
Tau	Microtubule-associated protein tau
*TARDBP*	*Transactive Response DNA-Binding Protein*
TAX1BP1	Tax1-Binding Protein 1
*TBK1*	*TANK-Binding Kinase 1*
TDP-43	Transactive response DNA-Binding Protein 43
TMEM192	Transmembrane Protein 192
TNF	Tumor Necrosis Factor
UBA	Ubiquitin-Associated Domain
UBD	Ubiquitin-Binding Domain
ULD	Ubiquitin-Like Domain
ULK1	Unc-51-Like autophagy-activating Kinase 1
*VCP*	*Valosine Containing Protein*
VPS	Vacuolar Protein Sorting
WDR41	WD Repeat Containing Protein 41
